# Nutritional strategies in kidney transplantation: optimising outcomes beyond surgery

**DOI:** 10.3389/ti.2026.16322

**Published:** 2026-09-24

**Authors:** Lara Caldiroli, Paolo Molinari, Simone Vettoretti, Anna Regalia, Simona Verdesca, Giuseppe Castellano, Maurizio Gallieni, Carlo Alfieri

**Affiliations:** 1 Department of Nephrology, Dialysis and Renal Transplantation, Fondazione IRCCS Ca’ Granda Ospedale Policlinico, Milan, Italy; 2 Department of Clinical Sciences and Community Health, University of Milan, Milan, Italy; 3 Ospedale Luigi Sacco-Polo Universitario, Milan, Italy

**Keywords:** immunosuppression, ketogenic diet, kidney transplantation, mediterranean diet, metabolic complications

## Abstract

Although kidney transplantation (KTx) remains the optimal therapy for end-stage kidney disease, long-term graft survival and patient outcomes continue to be limited by cardiometabolic complications and chronic allograft dysfunction. Nutritional management has evolved from supportive care to a core component of post-transplant follow-up. The early post-transplant phase is marked by catabolism, high-dose immunosuppression, and metabolic instability, leading to increased protein requirements but also potential susceptibility to hyperfiltration injury. In the stable post-transplant phase, a moderate protein intake similar to that recommended for chronic kidney disease (CKD) may support graft longevity, although evidence in kidney transplant recipients (KTRs) remains limited and inconsistent. Plant-forward dietary models, including Mediterranean and DASH patterns, consistently improve cardiometabolic risk, inflammation, oxidative stress, and dietary acid load, with potential benefits for graft outcomes. Predominantly plant-based diets may further reduce uremic toxin generation and improve mineral metabolism, though careful monitoring for micronutrient deficiencies is required. Low-protein approaches, such as PLADO, remain largely theoretical in transplant populations, while ketogenic or very low-carbohydrate diets raise concerns regarding acid–base balance, renal hemodynamics, and long-term tolerability. Overall, personalised nutritional strategies tailored to graft function, immunosuppression, and metabolic profile are essential to optimise long-term transplant health.

## Introduction

Kidney transplantation is the preferred treatment for end-stage kidney disease, offering superior survival and quality of life compared to chronic dialysis, but long-term outcomes are limited by metabolic complications, cardiovascular disease, and chronic allograft dysfunction.

[1] Nutrition, once considered ancillary, is now recognized as a pivotal determinant of graft and patient outcomes [1–3].

In the immediate post-transplant period, high-dose immunosuppression and catabolic stress necessitate adequate protein intake to maintain nitrogen balance and support lean mass. The recommended protein intake during this phase is typically 1.0–1.3 g/kg/day, as supported by recent reviews and observational studies [4–6]. However, excessive protein intake may induce glomerular hyperfiltration and accelerate graft injury, so intake above 1.4 g/kg/day is generally not advised for prolonged periods [4, 5]. During stable phases, moderate protein restriction (0.8–1.0 g/kg/day) may be beneficial for graft longevity, though evidence in transplant recipients is limited and sometimes conflicting [4, 7, 8]. For those with chronic allograft dysfunction or reduced eGFR (<25 mL/min), lower protein targets (0.55–0.60 g/kg/day) may be considered [4].

Beyond macronutrient targets, dietary patterns such as the Mediterranean and Dietary Approaches to Stop Hypertension (DASH) diets are associated with reduced cardiovascular risk and improved graft outcomes, emphasizing plant-based foods, reduced processed meats, and lower sodium intake [1, 7, 9, 10]. These patterns may also beneficially modulate the immune system and gut microbiome [3, 11].

In summary, nutritional management in kidney transplant recipients should be individualized, with attention to phase-specific protein requirements, overall dietary patterns, and metabolic risk factors, as supported by current evidence [1–12].

## Methods

This manuscript is a narrative review summarising the current evidence on nutritional strategies for kidney transplant recipients. A non-systematic literature search was performed using the PubMed/MEDLINE, Scopus and Google Scholar databases to identify studies published up to January 2026. Search terms included combinations of the following: “kidney transplantation,” “kidney transplant recipients,” “nutrition,” “diet,” “protein intake,” “Mediterranean diet,” “DASH diet,” “plant-based diet,” “vegetarian diet,” “ketogenic diet,” “microbiota,” and “allograft function.” Priority was given to clinical practice guidelines, randomised controlled trials, cohort studies, meta-analyses and high-quality narrative reviews published in English. This review aimed to provide an overview of emerging dietary approaches and nutritional management strategies in kidney transplantation from a clinical perspective, rather than conducting a formal systematic review or meta-analysis.

### Pre-transplant and peri-transplant nutritional optimization: prehabilitation and ERAS-based approaches

Nutritional management in kidney transplantation should not only begin after surgery. The pre- and peri-transplant periods are critical for identifying malnutrition, sarcopenia, obesity, frailty, micronutrient deficiencies and metabolic risk factors, which can affect surgical recovery and long-term transplant outcomes [1–4]. In other surgical populations, multimodal prehabilitation and Enhanced Recovery After Surgery (ERAS) protocols combining nutritional optimisation, physical exercise, anaemia correction, glycaemic control and patient education have been associated with a shorter hospital stay, fewer postoperative complications, faster functional recovery and improved survival [5–8].

However, direct evidence remains limited in kidney transplant candidates. Available studies suggest that poor nutritional status, sarcopenia, frailty and obesity prior to transplantation are associated with delayed graft function, infectious complications, prolonged hospitalisation, cardiovascular events and reduced patient and graft survival [9–14]. Therefore, pre-transplant nutritional education may be clinically relevant, particularly when integrated into multidisciplinary care involving nephrologists, nutritionists, transplant surgeons and rehabilitation specialists [1, 15]. Key objectives include optimising protein-energy status, avoiding protein-energy wasting in dialysis patients, managing weight in obese patients, correcting vitamin and mineral deficiencies, restricting sodium for blood pressure control, and counselling on food safety and metabolic risk after transplantation [1, 15–17].

Peri-transplant nutrition should aim to minimise catabolism and support wound healing, early mobilisation and recovery [6–8]. Although ERAS pathways are being adopted more widely in solid-organ transplantation, there is still insufficient standardisation of kidney-transplant-specific nutritional protocols [1, 3]. Currently, recommendations are largely extrapolated from general surgery, chronic kidney disease (CKD), dialysis, and transplant observational studies, rather than from adequately powered randomised trials in kidney transplant recipients [1, 3].

Future studies should evaluate whether structured pre-transplant nutritional education and multimodal prehabilitation programmes can improve clinically meaningful outcomes. These outcomes include postoperative complications, delayed graft function, length of hospital stay, readmissions, reversal of frailty, sarcopenia, quality of life, graft function trajectory and long-term patient survival [1, 3, 18].

### Dietary approaches for kidney transplant recipients

Over the past decade, several dietary patterns have grown in popularity due to their potential to reduce cardiovascular risk, exhibit anti-inflammatory properties, and promote weight loss [13]. Among these, plant-based diets and low-carbohydrate or ketogenic diets have received considerable attention (Supplementary Tables S1, S2).

#### Plant-based diets

In earlier decades, it was generally assumed that animal-derived proteins contain all the essential amino acids that the human body cannot produce in sufficient quantities (classifying them as proteins of high biological value), and they are more effective at maintaining the body’s protein pool due to their higher content of sulphur-containing amino acids [14, 15]. However, increasing interest in plant-based dietary approaches has challenged this paradigm. Diets composed primarily or exclusively of plant-derived foods are now considered feasible in populations with chronic diseases, including CKD [16]. A recent study demonstrated that the risk of incident CKD in the general population was reduced by 4% for each additional 0.1 g/kg/day of plant protein intake [14, 17]. Within nephrology, plant-based dietary patterns uniquely allow for the implementation of very low protein regimens (0.4 g/kg body weight/day) [1]. Compared with animal-based diets, plant-based diets provide higher amounts of fiber and alkali, which contribute to the improved correction of CKD-associated metabolic acidosis and the prevention of intestinal dysbiosis [3, 14]. It is important to note that plant-based diets and veganism are not synonymous. Rather, plant-based diets represent a spectrum of dietary models in which the majority of protein intake is derived from plant sources. Examples include the Mediterranean diet, the Dietary Approaches to Stop Hypertension (DASH) diet, and the PLADO diet. Due to their favorable metabolic effects, recent clinical practice guidelines on nutrition in CKD have endorsed plant-based dietary patterns. While both sets of guidelines acknowledge the limited evidence available to recommend one specific protein source, the KDOQI guidelines do not rule out plant-based diets, and the KDIGO guidelines explicitly recommend them [1, 14, 18].

##### Mediterranean diet

The Mediterranean diet is a dietary pattern that is primarily cardioprotective and anti-inflammatory. It originates from the traditional eating habits of people living in coastal regions of the Mediterranean. It is characterised by a high consumption of extra-virgin olive oil, vegetables, legumes and whole grains, as well as a moderate intake of fish and wine. Overall, it involves moderate protein intake of around 0.8 g/kg/day without being specifically designed as a low-protein regimen. [19]. Robust evidence supports its role in reducing the risk of type 2 diabetes, cardiovascular disease and all-cause mortality [20].

Adherence to the Mediterranean diet is associated with multiple metabolic and vascular benefits, including reductions in inflammation, oxidative stress, endothelial dysfunction, metabolic acidosis, hyperlipidemia, hyperglycemia and elevated blood pressure [4, 9, 10, 21, 22]. In both the general population and kidney transplant recipients (KTRs), higher Mediterranean Diet Scores correlate with improved renal outcomes [4, 23]. In a large multi-ethnic cohort of 3,298 individuals followed for 15 years, higher adherence was associated with approximately 50% lower odds of incident eGFR <60 mL/min/1.73 m^2^ [23]. Similarly, among 632 KTRs, adherence to the Mediterranean diet was protective against kidney function decline and graft loss, with stronger effects observed in patients with proteinuria [4].

The renoprotective effects of the Mediterranean diet likely arise from the modulation of several interconnected pathways, including oxidative stress, systemic inflammation, endothelial function, dietary acid load, lipid metabolism, protein intake and glycemic control. High consumption of fruits and vegetables increases antioxidant and dietary fiber intake, while a greater proportion of omega-3 fatty acids contributes to reductions in oxidative stress and inflammation [4, 24–26]. Low meat intake combined with high fruit and vegetable consumption improves acid–base balance [4, 27], and a lower dietary acid load is associated with reduced CKD risk [28].

The largely plant-based protein profile of the Mediterranean diet may also reduce proteinuria. Plant proteins, which have lower bioavailability and acidogenic potential, may induce less glomerular hyperfiltration and intraglomerular pressure than animal proteins, thereby reducing renal hemodynamic stress. Typical protein intake approximates 0.8 g/kg/day, mainly from non-red and non-processed sources [22]. Additionally, plant proteins are associated with a lower endogenous acid load and reduced sulphur and phosphorus amino acid content, potentially limiting tubular injury and hyperfiltration [3].

Finally, the diet’s high content of monounsaturated fats and polyphenols, and low saturated fat intake, further contributes to its protective effects [29–32]. In KTRs, improvements in oxidative status, acid–base balance and glomerular hemodynamics have been documented [4, 9, 10, 21–23, 29, 33]. Overall, the Mediterranean diet emerges as a valuable strategy to support general health and potentially long-term graft survival in KTRs.

##### Dietary approaches to stop hypertension (DASH) diet

The Dietary Approaches to Stop Hypertension (DASH) diet was originally developed by the US National Institutes of Health to treat or prevent hypertension. The DASH diet primarily targets sodium reduction and blood pressure control by encouraging the consumption of fruits, vegetables and low-fat dairy products, while reducing saturated fat intake and maintaining moderate protein consumption. It does not specifically promote a plant-based diet.

In the United States, the average sodium intake exceeds 3,400 mg per day, surpassing the maximum recommended intake of 2,300 mg per day set out in the 2015–2020 Dietary Guidelines [34]. Excess sodium intake has been linked to hypertension, cardiovascular events and, in experimental models, to the promotion of chronic allograft rejection [34]. Clinical evidence demonstrates that the DASH diet significantly lowers blood pressure, total cholesterol and low-density lipoprotein cholesterol levels [35]. In an 8-week controlled feeding trial, the DASH diet reduced systolic and diastolic blood pressure by 5.5 mmHg and 3.0 mmHg respectively compared to standard diets [36]. The DASH diet remains one of the most effective dietary strategies for blood pressure control [37].

Hypertension affects 50%–80% of adult KTRs, contributing to shorter graft survival and increased cardiovascular morbidity and mortality [38]. Thus, the DASH diet is a particularly relevant intervention for this population. In a prospective cohort study of 632 KTRs, greater adherence to the DASH diet was associated with a lower risk of decline in kidney function (hazard ratio: 0.95; P = 0.008) and reduced all-cause mortality (hazard ratio: 0.95; P = 0.01) [7]. Similar renal and cardiovascular benefits have been observed in other studies [9, 10, 39], which are likely to be mediated through favorable effects on serum lipids, blood pressure, insulin resistance, inflammation, oxidative stress, arterial stiffness and endothelial function. In summary, the DASH diet provides a robust, evidence-based approach to restricting sodium and controlling blood pressure in KTx patients.

##### Vegetarian and vegan diets

Vegetarian diets, historically adopted for religious, cultural or ethical reasons, are increasingly promoted for health and disease prevention. Evidence suggests that reducing or eliminating meat intake may attenuate kidney disease progression [9, 10, 40]. Vegetarian diets include heterogeneous patterns, from lacto-ovo vegetarian to vegan diets, and do not inherently guarantee nutritional quality, as processed foods may still be consumed [40].

In kidney transplant recipients, protein source and quantity critically influence graft survival. High protein intake, especially from animal sources, induces afferent arteriolar dilation, glomerular hyperfiltration and intraglomerular hypertension, promoting fibrosis and progressive renal injury [40–42]. Protein restriction reduces hyperfiltration and stabilizes kidney function [42]. Data from chronic kidney disease populations show that low-protein diets delay dialysis initiation and slow progression to end-stage kidney disease [43, 44], whereas high-protein and red-meat–based diets are linked to faster kidney function decline and increased microalbuminuria [45, 46].

Plant-based diets improve mineral metabolism and acid–base balance. Plant phosphorus has lower bioavailability, resulting in reduced serum phosphate and FGF23 levels associated with better graft outcomes [9, 40, 47–51]. Plant potassium generally has milder effects due to enhanced fecal excretion and alkalinisation [9, 40, 52, 53]. High fiber intake reduces uremic toxins and inflammation [54–56]. Nutritional risks include vitamin B12 and iron deficiency, requiring monitoring [57]. Overall, well-planned plant-based low-protein diets may support long-term graft preservation [12, 58].

##### Low protein intake and plant dominant low protein diet (PLADO)

There is growing evidence that protein restriction slows chronic kidney disease progression [3, 59–62], although its impact on kidney transplantation outcomes remains uncertain. Dietary protein intake drives urea and nitrogenous waste production, increasing renal excretory burden. Protein restriction activates protective mechanisms, including downregulation of the renin–angiotensin system [3, 63, 64], reduced glomerular hemodynamic stress [59, 65, 66], limited renal hypertrophy [63], decreased ammoniagenesis [63] and lower metabolic activity [67]. Conversely, high-protein diets increase metabolic demand, promote renal vasodilatation and exacerbate intraglomerular pressure in transplant kidneys, leading to hyperfiltration and structural injury [42, 59].

Plant-focused low-protein dietary approaches, including the Plant-Dominant Low-Protein Diet (PLADO) and the Plant-Focused Nutrition in Diabetes (PLAFOND), have been proposed for CKD and diabetes management. The Plant-Dominant Low-Protein Diet (PLADO) is a structured, nephroprotective dietary strategy designed to reduce glomerular hyperfiltration and metabolic burden. It involves controlled protein restriction (0.6–0.8 g/kg/day) and a diet consisting predominantly of plant-derived protein sources (generally 50%–75%) [2, 68]. The Plant-Focused Nutrition in Diabetes (PLAFOND) model further emphasises high fiber intake, low glycemic index and adequate energy provision [69]. Although these diets may slow CKD progression, evidence supporting benefits or safety in kidney transplant recipients is lacking.

Evidence on protein intake after transplantation is conflicting. Low intake may preserve graft function, whereas higher intake supports nitrogen balance, muscle mass, fatigue and quality of life [2, 4, 6, 70, 71].

#### Low-carb diets

Metabolic complications are common after kidney transplantation, and dietary strategies have been explored to limit weight gain, hypertension, dyslipidemia and insulin resistance [13]. Immunosuppressive therapies, especially glucocorticoids, promote hyperglycemia and obesity [72–76]. Adequate glycemic control is clinically relevant because it is associated with slower kidney disease progression [77–79].

Ketogenic diets have gained interest for metabolic control, improving satiety, weight reduction and insulin sensitivity, while reducing inflammation [80–82]. A ketogenic diet usually limits carbohydrates to 30–50 g/day, inducing nutritional ketosis through glycogen depletion and ketone body production [83, 84]. Ketone bodies provide energy for several organs, including kidneys, during carbohydrate restriction.

Nutritional ketosis is defined by ketone levels of 0.5–3 mmol/L [85], whereas therapeutic ketosis reaches higher concentrations [86]. Keto-adaptation involves mitochondrial and enzymatic upregulation in oxidative tissues [85, 87]. Importantly, nutritional ketosis differs from diabetic ketoacidosis, as pH and glucose remain normal.

In kidney transplant recipients, evidence on ketogenic diets is limited and controversial. Potential concerns include acid load, hyperkaliemia, protein catabolism and graft injury, although early CKD data suggest preserved pH during nutritional ketosis [85, 88]. Reported adverse effects include fatty liver and insulin resistance [40, 89, 90]. Data in transplant populations are scarce, heterogeneous and insufficient to define safety or graft benefit. Short-term use under specialist supervision appears feasible for obesity, but long-term advantages resemble those of Mediterranean-style diets [91].

High reliance on processed ketogenic products may be inappropriate for transplant recipients. Moreover, long-term adherence is difficult, and carbohydrate quality may be more relevant than quantity [92, 93]. Diets rich in whole, high-fiber carbohydrates show superior cardiovascular and metabolic outcomes compared with ketogenic diets [94]. In the post-transplant setting, high-quality carbohydrates may reduce post-transplant diabetes and obesity risk [95, 96]. Therefore, balanced dietary patterns emphasizing carbohydrate quality may better address metabolic complications after transplantation.

### Dietary protein intake: implication for kidney allograft survival and function

There is currently insufficient evidence to determine the optimal quantity or type of dietary protein for KTRs, so formal recommendations cannot yet be established. Nevertheless, certain patient- and transplant-related factors may inform the individualisation of protein intake.

The 2020 KDOQI Clinical Practice Guideline for Nutrition in CKD recommends a low-protein diet of 0.55–0.60 g/kg/day or a very low-protein diet of 0.28–0.43 g/kg/day, supplemented with keto acid or amino acid analogues, for adults without diabetes. For adults with CKD and concomitant diabetes, an intake of 0.6–0.8 g/kg/day of protein is considered appropriate. It is notable that no specific dietary protein intake targets are currently provided for adult kidney transplant recipients [1].

#### The net state of immunosuppression and dietary protein requirements in kidney transplant recipients

The immunosuppressive status of KTRs fluctuates over time. During the immediate post-transplant period and when treating acute rejection, escalating the use of immunosuppressive agents — particularly high-dose corticosteroids — can accelerate protein catabolism and lead to muscle wasting [2, 97]. To counteract muscle loss, protein intake should be increased [2].

Evidence from two key studies has provided insights into protein needs (Table 1). A prospective cohort study of KTRs after deceased donor kidney transplantation (DDKT) found that patients consuming a low-protein diet had higher protein catabolic rates than those with higher protein intake [6]. Similarly, a randomised controlled trial involving non-diabetic KTRs found that low protein intake was associated with negative nitrogen balance and muscle mass loss, whereas higher protein intake resulted in positive nitrogen balance and muscle gain [2]. These studies suggest that an intake of at least 1.0–1.3 g/kg/day of protein may help preserve lean mass during the early post-transplant phase.

**TABLE 1:** Suggested protein intake in kidney transplant recipients across different clinical phases.

Clinical phase	Immunosuppressive status	Suggested protein intake	Evidence strength
Immediate post-transplant (0-1 month, high-dose corticosteroids) [11, 12, 17]	Escalated immunosuppression, high-dose steroids	1.0–1.3 g/kg/day to maintain nitrogen balance and prevent muscle wasting	Higher intake (≥1.4 g/kg/d; mean ∼1.6–1.8) associated with better early eGFR, hemoglobin, lipids and no increase in short-term complications or infections Suggested evidence strength: Low–moderate (retrospective cohorts, observational)
Early stable post-transplant (1-6 months, low or stable steroid dose) [13]	Reduced/stable immunosuppression	≥0.72 g/kg IBW/day to avoid skeletal muscle loss	≥0.72 g/kg IBW/day predicted to prevent net skeletal muscle loss at 12 months. Several cohorts link higher intake (∼1.0–1.1 g/kg/d) with better fatigue, QoL, and survival proxies Evidence strength: Low–moderate (observational, modeling)
Medium -long term post -transplant [13]	Reduced/stable immunosuppression	≥0.72 g/kg IBW/day to avoid skeletal muscle loss	Reviews suggest 0.8–1.0 g/kg/d for stable KTRs, with cautious trial of 0.6–0.8 g/kg/d low-protein, plant-dominant patterns to mitigate hyperfiltration, if nutritional status is preserved Evidence strength: Low (narrative reviews, conflicting observational data)
Chronic allograft dysfunction/rejection [1]	Variable; inflammation may be present, corticosteroids often tapered	≥0.55–0.60 g/kg/day (low protein diet may improve glomerular permselectivity, but intakes <0.55 g/kg/day risk negative nitrogen balance)	Proposed range aims to slow progression while avoiding negative nitrogen balance; largely extrapolated from CKD LPD data and one crossover KTR nitrogen-balance study Evidence strength: Low (CKD RCTs, small transplant studies, extrapolation)
Failed allograft (return to dialysis) [15]	Immunosuppression generally minimized or stopped	1.0–1.2 g/kg/day (aligned with ESKD recommendations)	Aligns with ESKD guidelines and hemodialysis data, where ≥1.0–1.2 g/kg/d is recommended to prevent protein-energy wasting Evidence strength: Moderate (CKD/ESKD trials and guidelines; indirect for failed graft)

ESKD: end stage kidney disease; IBW:ideal body weight.

When steroid doses are tapered, the need for protein appears to be lower. A cross-sectional study indicated that a minimum intake of 0.72 g/kg/day may be sufficient 1 year after transplantation [2]. Conversely, in cases of chronic allograft dysfunction, the inflammatory environment may increase protein requirements. Data from a crossover RCT involving 14 KTRs with chronic rejection compared low (0.55 g/kg/day) and high (2.0 g/kg/day) protein diets. The low-protein group showed lower urinary protein and better glomerular permselectivity, though they only achieved a slightly positive nitrogen balance [98].

It is important to note that current nutritional recommendations for kidney transplant recipients are supported by relatively limited transplant-specific evidence and are often based on studies conducted in populations with chronic kidney disease. The unique physiological and metabolic characteristics of kidney transplant recipients, including chronic immunosuppression, altered inflammatory status, post-transplant metabolic complications and the presence of a functioning allograft, may limit the direct applicability of CKD-derived nutritional strategies. Consequently, adequately powered randomised controlled trials conducted specifically in kidney transplant populations are urgently needed to define optimal dietary patterns, protein and energy requirements, and long-term nutritional interventions capable of improving graft survival, cardiometabolic outcomes, sarcopenia and patient quality of life.

#### Dietary calorie-protein intake across post-transplant periods

In addition to immunosuppression, the post-transplant period itself influences protein requirements. In a 12-year prospective cohort of 43 KTRs, a low-protein intake (0.73 g/kg/day) was associated with stable GFR, whereas higher intake (1.40 g/kg/day) was associated with a significant decline [99].

However, evidence from observational studies remains inconsistent. In a cross-sectional analysis of 204 KTRs, protein intake >1.2 g/kg/day was associated with proteinuria [5], while a larger cohort of 625 KTRs showed no association with creatinine clearance [100]. It is important to note that excessively low protein intake may also be detrimental: in cohorts of 604 and 940 KTRs, lower protein intake was associated with an increased risk of graft loss and mortality [8, 101].

Overall, the current evidence base supports a moderate protein intake of 0.8–1.0 g/kg/day for the first three to twelve months after transplant, with the possibility of increasing this to 1.2–1.4 g/kg/day thereafter. Recommendations following graft failure are in line with the guidelines for end-stage kidney disease (ESKD) (0.55–0.6 g/kg/day) (Figure 1) [1].

**FIGURE 1:**
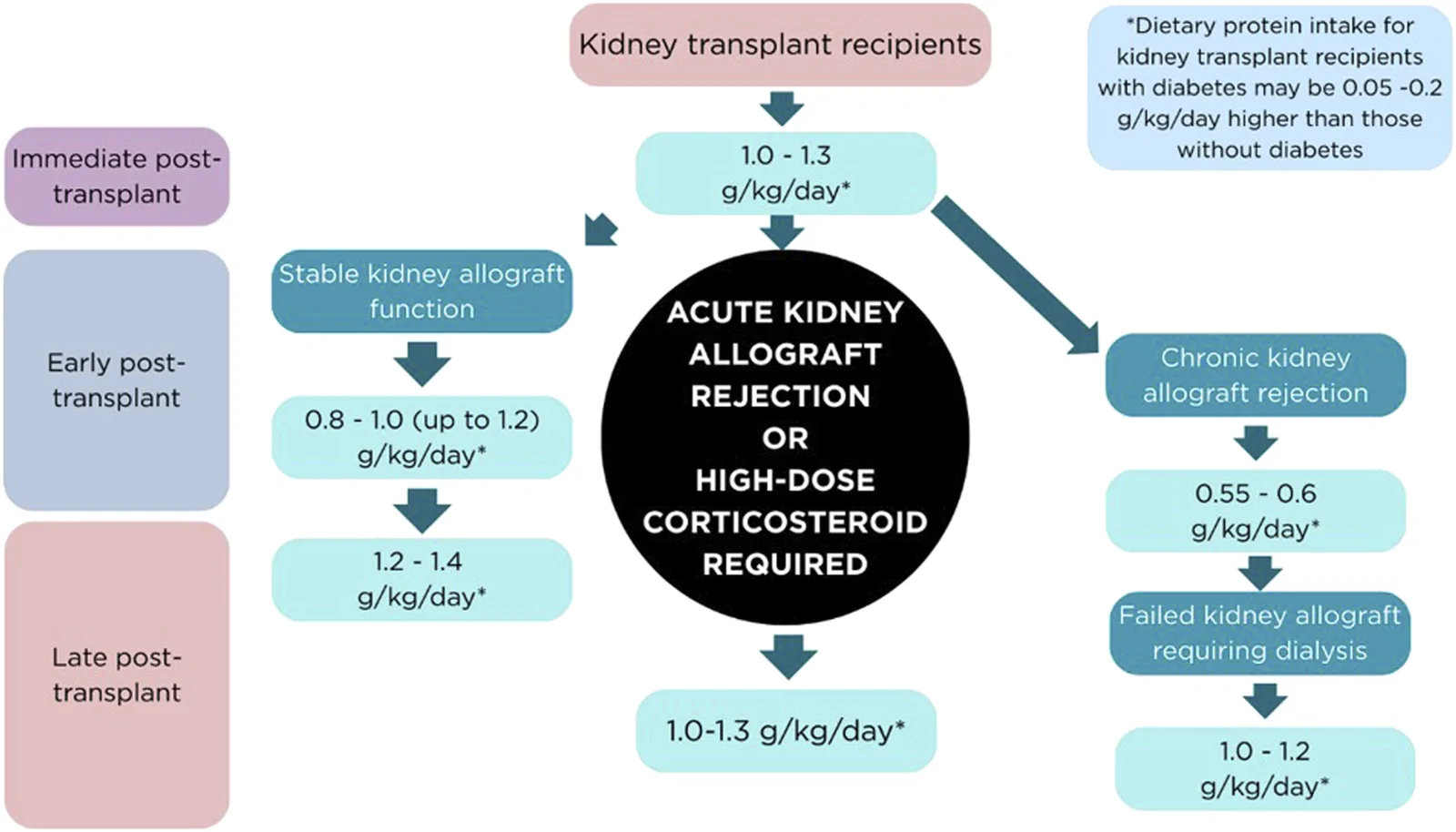
Protein intake in different post-RTx periods and among different clinical conditions.

Adequate energy intake is a critical component of nutritional management after kidney transplantation and should accompany protein prescriptions to maintain neutral nitrogen balance and prevent muscle catabolism [1, 2, 70]. In the immediate post-transplant period, calorie requirements generally increase due to surgical stress, exposure to corticosteroids, tissue repair and heightened metabolic demand [2, 6, 70]. Approximately 30–35 kcal/kg/day is commonly suggested as an energy intake during the early catabolic phase, whereas lower requirements (25–30 kcal/kg/day) may be appropriate during stable long-term follow-up, depending on factors such as age, physical activity, body composition, graft function, and metabolic comorbidities [1, 58]. It is important to note that inadequate caloric intake can lead to the breakdown of endogenous proteins and sarcopenia, even when protein intake appears sufficient [2, 6, 71]. Conversely, excessive calorie intake can lead to obesity, post-transplant diabetes mellitus, metabolic syndrome and cardiovascular complications. These remain major determinants of long-term graft and patient outcomes [13, 72, 95, 96].

### Comparison of international guidelines (ESPEN, KDOQI, KDIGO)

Compared with older, nutrient-restriction paradigms, today’s triad of ESPEN–KDOQI–KDIGO collectively pushes toward: early anabolic support; longer-term weight, blood pressure (BP), and glycemic control; and endorsement of healthy dietary patterns rather than transplant-unique restrictions. Collectively, these guidelines reflect a paradigm shift: from nutrient restriction to holistic, patient-centered dietary strategies (Table 2).

**TABLE 2:** comparison of the main features of different nutrition guidelines focusig on KTRs.

Guideline	Kidney transplant recipient (KTR) coverage	Key nutrition recommendations	Limitations
ESPEN (2021; 2024 update)	No specific guidance for stable KTR; transplant nutrition not addressed	Inpatient focus: energy/protein delivery, artificial nutrition routes, refeeding prevention	Gap in outpatient and post-transplant care; limited applicability beyond hospitalization
KDOQI (2020)	Explicitly includes patients with functioning kidney transplants	- Routine nutrition screening and assessment (validated tools, body composition) - Individualized protein/energy prescriptions by stage (higher in acute post-transplant, tailored for long-term) - Sodium restriction for BP control - Promotion of dietary patterns based on whole, minimally processed foods	Does not mandate a single protein target for all KTR; evidence for long-term outcomes still limited
KDIGO (2009–2022)	Scattered transplant-related recommendations across thematic guidelines	- Lifestyle and CV risk management integrated with immunosuppression - Pre-transplant risk factor optimization (e.g., obesity) - Post-transplant: BP control (standardized measurement, sodium moderation), weight management, PTDM prevention	No standalone nutrition CPG for KTR; lacks detailed macronutrient prescriptions

KTR: kidney transplant recipients; BP: blood pressure; CV: cardiovascular; PTDM: Post-transplant Diabetes Mellitus; CPG: clinical practice guidelines.

### Intestinal microbiota and nutritional regimens in kidney transplant patients

The gut microbiota is increasingly recognized as a key modulator of health and transplant outcomes. After transplantation, KTRs commonly develop dysbiosis, with reduced microbial diversity and depletion of short-chain fatty acids (SCFAs)-producing bacteria such as Faecalibacterium prausnitzii [102]. Immunosuppressive agents directly alter microbial composition and function [103]. This imbalance may impair gut barrier integrity and promote systemic inflammation [104].

Dietary modulation offers an effective strategy. High-fiber diets enhance microbial fermentation and SCFA production, which support epithelial integrity and improve tacrolimus bioavailability [11]. Butyrate specifically promotes regulatory T-cell differentiation [105]. Conversely, patterns high in red meat and refined sugars increase production of harmful metabolites such as trimethylamine N-oxide [106] (Figure 2).

**FIGURE 2:**
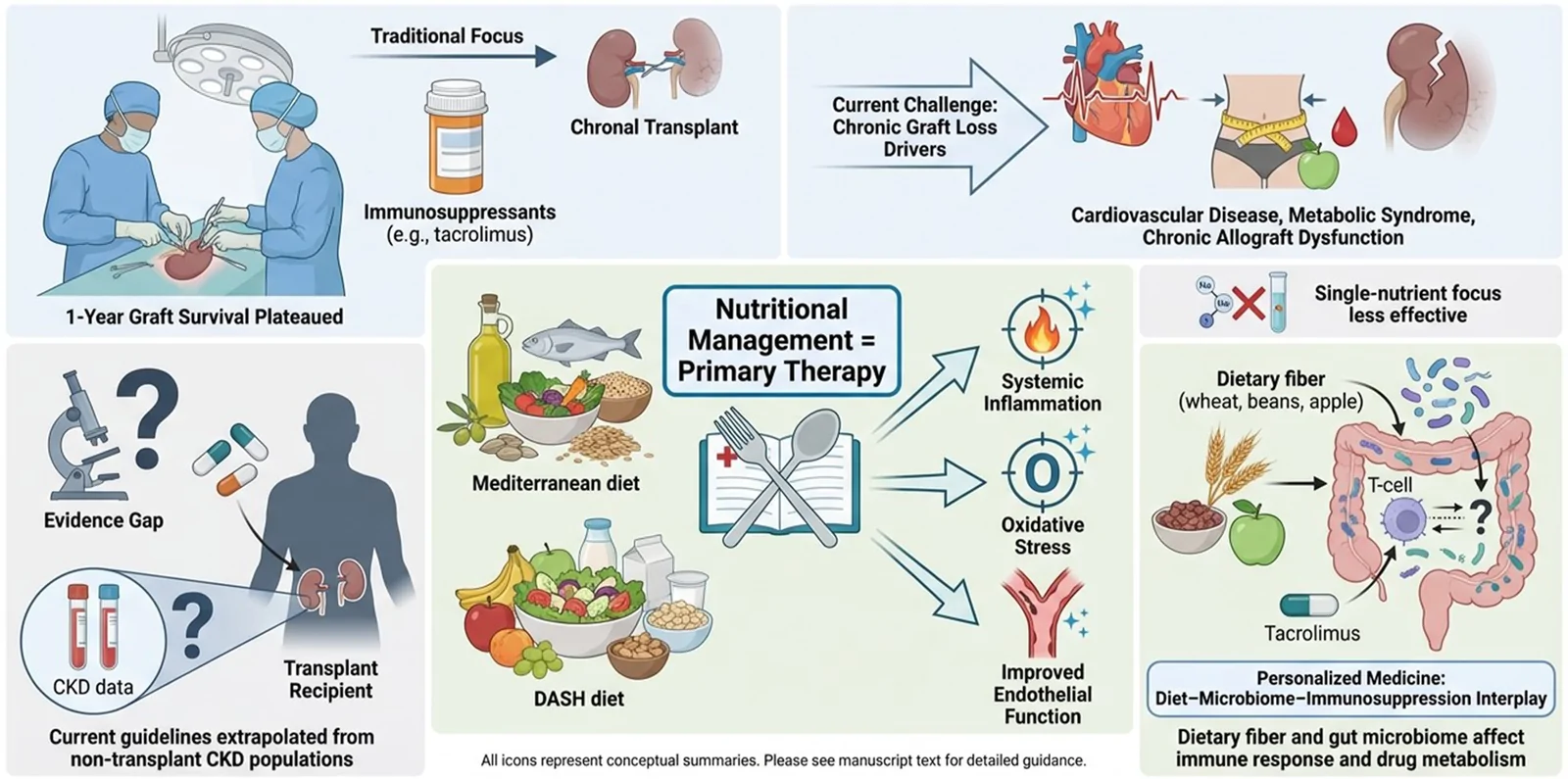
The evolving landscape of post-transplant nutrition: from immunosuppression to dietary-microbiome interplay.

#### Clinical recommendations and future directions

The following recommendations, derived from available and current evidences, provide a roadmap for the longitudinal care of KTRs, emphasizing a multidisciplinary, phase-specific approach.

### Macro-structural dietary recommendations

- Prioritizing the Mediterranean Framework: For the stable KTR, the Mediterranean diet should be the default recommendation. Its emphasis on extra-virgin olive oil, high fiber, and plant-derived proteins offers a synergistic effect that addresses the leading cause of death in this population: cardiovascular disease.
- The DASH Alternative: In patients where hypertension is the primary driver of allograft injury, the DASH diet provides a structured, evidence-based approach to sodium restriction while ensuring nutritional adequacy.
- Carbohydrate Quality and Metabolic Syndrome: Given the high incidence of post-transplant diabetes mellitus (PTDM), clinicians should move away from simple carbohydrate restriction. Instead, the focus should be on carbohydrate quality—prioritizing whole grains and low-glycemic-index foods to counteract the metabolic disturbances induced by glucocorticoids and calcineurin inhibitors.

### The precision protein prescription

Protein intake must be tailored to the “chronological age” of the transplant and the patient’s metabolic demands.

- The Early Catabolic Window (0–3 Months): To counteract surgical stress and steroid-induced muscle wasting, an intake of 1.0–1.3 g/kg/day is recommended.
- The Maintenance Phase (3–12 Months): Transition to 0.8–1.0 g/kg/day. Clinicians must be vigilant to ensure intake does not fall below 0.72 g/kg/day to avoid sarcopenia, which is independently associated with poor outcomes.
- The Dysfunction Phase (Chronic Allograft Nephropathy): In the setting of declining eGFR, moderate restriction to 0.55–0.60 g/kg/day may be protective, provided the patient is not in a state of chronic inflammation.

### Microbiome-targeted interventions

Fiber as an Immunomodulator: Clinicians should encourage high dietary fiber intake (25–30g/day) not just for gastrointestinal health, but as a strategy to produce short-chain fatty acids (SCFAs). SCFAs, specifically butyrate, may support immune tolerance and protect the gut-vascular barrier.

To advance the field of transplant nutrition, future research must prioritize large-scale, transplant-specific randomized controlled trials to evaluate the long-term impact of plant-dominant dietary patterns, such as the PLADO diet, on eGFR slopes and graft longevity. Additionally, rigorous investigations are needed into the safety and efficacy of nutritional ketosis as a targeted intervention for refractory post-transplant obesity, specifically assessing its potential to improve metabolic markers without precipitating metabolic acidosis. There is also a compelling need for precision nutrition strategies that utilize microbial profiling to personalize fiber and protein intake based on the individual’s unique gut environment.

Ultimately, while the technical success of a kidney transplant begins in the operating room, its long-term preservation is sustained in the kitchen; transitioning to personalized, pattern-based nutritional therapy is therefore essential to ensuring that the gift of a transplant lasts a lifetime (Figure 3.).

**FIGURE 3:**
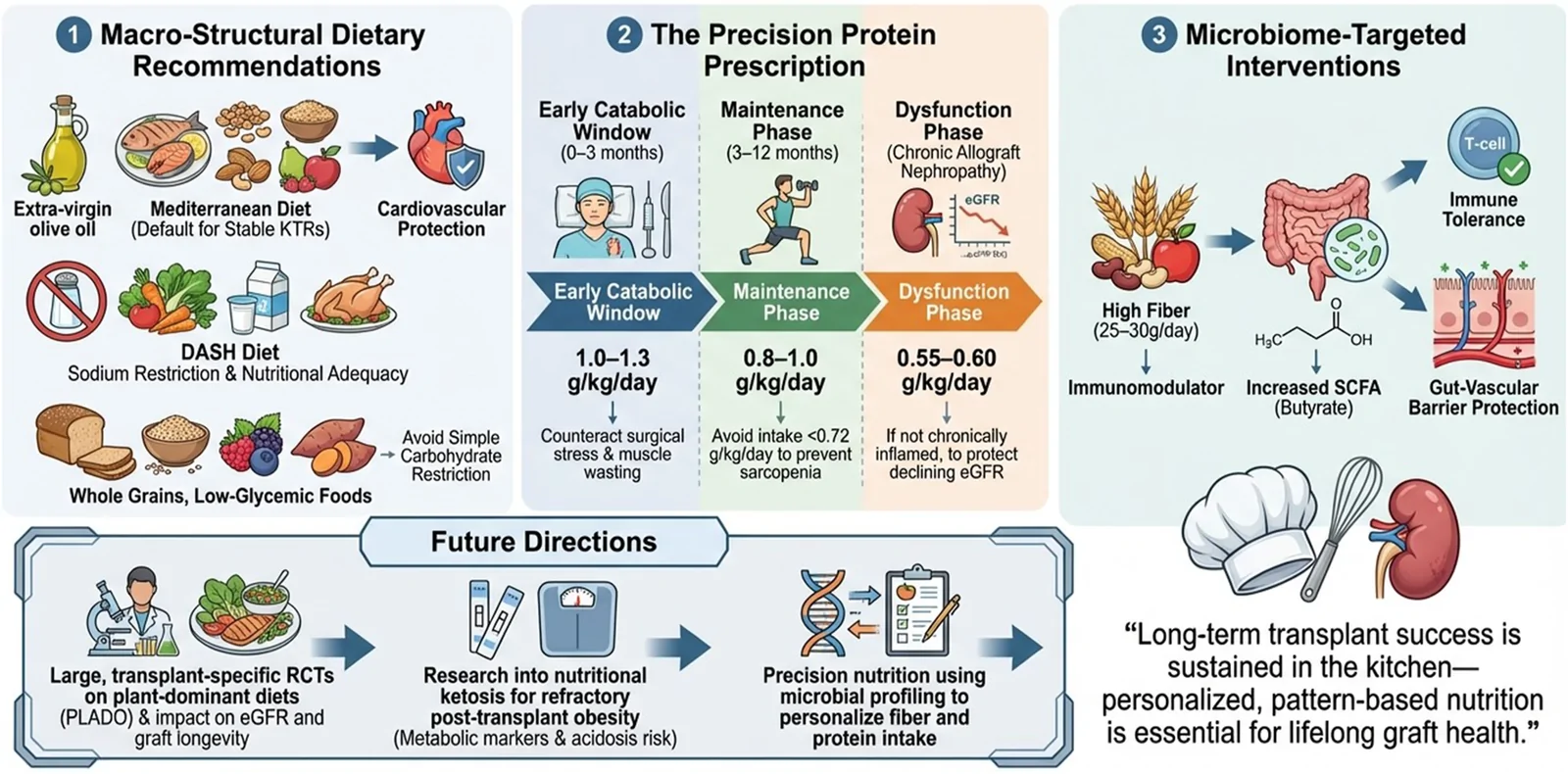
The clinical roadmap for longitudinal care of KTRs phase-specific and multidisciplinary nutrition.

## Conclusions: a new paradigm in transplant nephrology

The historical focus of transplant medicine has traditionally centered on the refinement of surgical techniques and the optimization of immunosuppressive regimens to prevent acute rejection. However, as 1-year graft survival rates have plateaued at high levels, the challenge has shifted toward mitigating the chronic drivers of graft loss: cardiovascular disease, metabolic syndrome, and chronic allograft dysfunction.

This review highlights a significant transition in clinical thought: nutritional management is no longer a secondary support mechanism but a primary therapeutic intervention.

Data from literature underscores that focusing on single nutrients (e.g., sodium or protein) in isolation is less effective than adopting comprehensive dietary patterns. The Mediterranean and DASH diets emerge as the most robust frameworks because they address the multi-faceted nature of post-transplant health—simultaneously targeting systemic inflammation, oxidative stress, and endothelial dysfunction.

While the biological rationale for plant-based and low-protein diets is strong—centering on the reduction of glomerular hyperfiltration and acid load—this review identifies a critical “evidence gap.” Much of our current practice relies on the extrapolation of data from non-transplant CKD populations. The unique physiology of the transplant recipient, characterized by pharmacological immunosuppression and a solitary functioning kidney, necessitates a dedicated body of research.

One of the most compelling frontiers identified is the interaction between nutrition and the gut microbiome. The realization that dietary fiber can modulate the immune response and potentially alter the metabolism of drugs like tacrolimus opens a new chapter in personalized transplant medicine. This suggests that the “net state of immunosuppression” is not merely a product of drug dosage, but a complex interplay between pharmacology and the intestinal environment (Figure 2).

The evidence supporting nutritional interventions in kidney transplant recipients is constrained by substantial methodological limitations. Most available studies are observational, retrospective, or conducted in relatively small cohorts, which restricts statistical power, external validity, and causal inference. Dietary intake is typically assessed using self-reported questionnaires, introducing recall and measurement bias. Residual confounding is also considerable, as healthier dietary patterns often cluster with other favourable lifestyle behaviours. In addition, transplant populations are highly heterogeneous with respect to immunosuppressive regimens, graft function, and comorbid conditions. Finally, many nutritional recommendations—particularly regarding plant-based and low-protein diets—are extrapolated from chronic kidney disease cohorts rather than derived from randomized trials in transplant recipients, highlighting the urgent need for adequately powered, transplant-specific studies.
