# Supplementary material for: Nutritional strategies in kidney transplantation: optimising outcomes beyond surgery
Source: Transpl Int. 2026 Sep 24;39:16322. doi: 10.3389/ti.2026.16322 (PMC13645717; doi:10.3389/ti.2026.16322)
Supplement: Supplementary file 1 [file Table1.docx]

**Table S1:** Comparison of evidence strength of different dietary patterns in kidney transplant patients

| **Dietary pattern / intervention** | **Evidence in KTRs** | **Evidence extrapolated from CKD/general population** | **Overall level of evidence** |
| --- | --- | --- | --- |
| **Mediterranean diet** | Multiple observational cohorts in KTRs (e.g., n=632) associated with lower graft decline and graft loss risk [25] | Large cohort studies and meta-analyses demonstrating cardiovascular and renal protection [22,26-28] | **Moderate-to-strong** |
| **DASH diet** | Prospective cohort in KTRs (n=632) showing reduced eGFR decline and mortality [44] | Extensive hypertension and cardiovascular outcome trials in general/CKD populations [40-42] | **Moderate** |
| **Plant-based / vegetarian diets** | Limited transplant-specific observational data; mechanistic rationale for lower acid load and reduced hyperfiltration [46] | Strong CKD literature supporting renal and metabolic benefits [3,19,57] | **Moderate (mainly extrapolated)** |
| **Low-protein diets (PLADO/PLAFOND)** | Very limited KTR-specific data; small studies suggest reduced hyperfiltration [108,109] | Multiple CKD trials and meta-analyses demonstrating slower CKD progression [49,50,67-69] | **Low-to-moderate (mostly extrapolated)** |
| **High protein intake in early post-transplant phase** | Small metabolic and cohort studies supporting preservation of lean mass and nitrogen balance [75,78,80] | Supported by catabolic physiology literature and ESKD nutrition guidelines | **Moderate** |
| **Ketogenic / low-carbohydrate diets** | Sparse and heterogeneous transplant-specific evidence; no long-term outcome studies [46] | Emerging CKD and metabolic disease literature with conflicting safety data [87,93] | **Low / hypothesis-generating** |
| **High-fiber / microbiota-targeted interventions** | Small feasibility studies and mechanistic evidence in KTRs [11,117] | Growing experimental and CKD microbiome literature [61,116,119] | **Low-to-moderate** |

**Note:** KTRs: kidney transplant recipients; CKD: Chronic Kidney Disease

**Table S2:** main characteristics of principal dietary regimens.

| **Dietary pattern** | **Primary therapeutic goal** | **Typical protein intake** | **Degree of plant dominance** | **Sodium restriction focus** | **Distinctive features** | **Potential role in KTRs** |
| --- | --- | --- | --- | --- | --- | --- |
| **Mediterranean diet** | Cardiovascular and anti-inflammatory protection | ~0.8 g/kg/day | Moderate-to-high | Moderate | Extra-virgin olive oil, fish, legumes, whole grains, moderate wine intake | Broad cardiometabolic protection and long-term graft preservation |
| **DASH diet** | Blood pressure and cardiovascular risk reduction | Moderate (~0.8–1.0 g/kg/day) | Moderate | Strong | Structured sodium restriction, high fruit/vegetable intake, low-fat dairy emphasis | Particularly useful in hypertensive KTRs |
| **PLADO (Plant-Dominant Low-Protein Diet)** | Reduction of hyperfiltration and metabolic kidney stress | 0.6–0.8 g/kg/day | High (≥50–75% plant protein) | Variable/moderate | Explicit low-protein structured regimen designed for CKD nephroprotection | Potential role in chronic allograft dysfunction; evidence still limited |
| **PLAFOND (Plant-Focused Nutrition in Diabetes)** | CKD and metabolic/diabetes management | 0.6–0.8 g/kg/day | High | Moderate | Plant-focused low-protein dietary model with emphasis on glycemic control and fiber intake | Potential benefit in KTRs with PTDM/metabolic syndrome |
| **Ketogenic / low-carbohydrate diet** | Weight loss and glycemic control | Often moderate-to-high | Low | Variable | Severe carbohydrate restriction with nutritional ketosis | Limited evidence; potential metabolic benefits but uncertain renal safety |

**Note:** KTRs: kidney transplant recipients; CKD: Chronic Kidney Disease
